# How patient participation was used to develop a questionnaire that is fit for purpose for assessing quality of life in severe asthma

**DOI:** 10.1186/s12955-018-0851-9

**Published:** 2018-01-27

**Authors:** Michael E. Hyland, Joseph W. Lanario, Jill Pooler, Matthew Masoli, Rupert C. Jones

**Affiliations:** 1 School of Psychology, University of Plymouth, Plymouth, PL4 8AA UK; 2 Plymouth Hospital’s NHS Trust, Plymouth, UK; 3 Peninsula School of Medicine and Dentistry, University of Plymouth, Plymouth, UK

**Keywords:** Assessment, Content validity, Questionnaire construction, Methodology, Patient reported outcome, Severe asthma

## Abstract

**Background:**

Previous research shows that existing asthma quality of life questionnaires fail to measure the burden of oral corticosteroids that can be used to treat severe asthma, and are therefore not fit for purpose for severe asthma according to the USA’s Federal Drug Authority’s (FDA) criteria for content validity. Patient input and documentation of that input is key to achieving content validity according to FDA guidelines. This paper describes the process of constructing a new questionnaire to measure the burden of asthma symptoms and burden of treatment in severe asthma, using criteria specified by the FDA.

**Methods:**

A draft severe asthma questionnaire (SAQ) was constructed using qualitative input from severe asthma patients who took part in an earlier study. The aim of this study was to improve that draft questionnaire using a further group of patients. In four iterative focus groups, 16 people with severe asthma completed the draft questionnaire, discussed the wording and structure and suggested changes that were incorporated into the final version.

**Results:**

The original intention to ask patients to identify whether problems were caused by asthma symptoms or side effects of medication was abandoned as the attribution of cause was found to be difficult and inconsistent. The recall period of 2 weeks was acceptable but fails to reflect the patients’ desire to express the variability of severe asthma. Patients suggested improvements to the wording of the draft questionnaire, including splitting some items in two, combining two items in one, and changes to some of the words in individual items and the response scale.

**Conclusions:**

The final version of the questionnaire was substantially different from one constructed using only qualitative reports from patients about the quality of life deficits of severe asthma. Patients make a valuable contribution to the questionnaire if they are asked to comment and improve an initial draft and where patients are treated as partners in the process of questionnaire construction, rather than only as a source of information to experts who construct the questionnaire.

## Background

Several asthma specific quality of life scales have been developed [1–3], and are used in clinical trials of asthma treatments. The development of these commonly used asthma specific scales occurred at a time before the publication of the USA’s Federal Drug Administration (FDA) recommendations for scale validity. The FDA recommendation is based on the concept of ‘fit for purpose’ where validity is relative to the population and use [4], and contrasts with earlier psychometric approaches [5]. Although these earlier scales may satisfy both the psychometric and FDA criteria for validity for mild or moderate asthma, none are fit for purpose for patients with severe asthma because they fail to capture some of the health-related quality of life deficits that characterise this particular group of patients [6], namely, those resulting from the effects of treatment burden [7].

The documentation provided by the FDA [4] proposed that questionnaire construction should be an iterative process involving: (a) concept definition, including a description of the population and the use to which the scale is put, (b) content validation where the items are constructed and evaluated using qualitative studies and (c) a construct and other validation using quantitative data. This paper describes the concept definition and content validation of a severe asthma quality of life scale that is fit for purpose as defined by the FDA.

### Concept definition

Severe asthma is defined as asthma that requires treatment with high dose inhaled corticosteroids plus a second controller and/or systemic corticosteroids to prevent it from becoming “uncontrolled” or that remains “uncontrolled” despite this therapy [8].

In contrast to mild or moderate asthma, two distinguishing features of severe asthma are (a) poor asthma control causing symptom variability over time including exacerbations that may require inpatient care and (b) an increased burden of side effects from medication. The concept of health-related quality of life in this instance must therefore include both types of deficit. The Severe Asthma Questionnaire (SAQ) is intended to measure is *the quality of life burden of asthma symptoms and the side effects of asthma medication as perceived by people who meet the criteria for severe asthma*. The scale is intended to be appropriate for use in clinical trials where different types of asthma treatment are compared, as well as providing a monitoring tool in clinical practice. In order to be useful in these contexts, the SAQ should enable patients to describe their health-related quality of life in a way that is meaningful to the patient, to be sufficiently short and easy to complete so as to reduce patient burden, and to have response scales that are sensitive to change.

The high burden of treatment in severe asthma, especially oral corticosteroids, is important because of the development of new biologic agents that, in addition to improving asthma control, can lead to a reduction in the use and hence side effects of oral corticosteroids. In an evaluation of the quality adjusted life years (QALY) one of these biologic agents, the National Institute for Care Excellence (NICE) noted that “some benefits of avoiding the adverse effects of oral corticosteroid use had not been fully captured in the QALY measure. P. 45” [9]. Frustration has been expressed by physicians about the process of evaluating the cost effectiveness of biologics [10] which they feel underestimates the benefit of these new treatments.

Although the primary intention is to have a single questionnaire that captures both the benefits and side effects of treatment in a single metric, there would be merit if these two aspects could be disambiguated so as to provide information for clinical practice. Thus, there is an extension to the concept defined above, namely to provide independent assessments of the burden of asthma symptoms and the burden of side effects of asthma treatment. This extension was based on the assumption that that patients could identify two components of health-related quality of life: that caused by the symptoms of the disease, and that caused by the symptom side effects of treatment.

### Content validity

The FDA documentation specifies that qualitative studies should ensure that the “domains of an instrument are appropriate and comprehensive relative to its intended measurement concept, population, and use” [4]. We have previously documented the domains of quality of life deficit in severe asthma, identifying two broad domain categories, (activities and emotional impact) and 11 more specific domains (hospitalisation, depression, irritability, sleep, hunger, weight, skin, gastric, pain, disease anxiety, and medication anxiety.) For each of these domains the proportion of patients experiencing the problem was identified. This earlier research can be used to identify the domains of a questionnaire [6].

The FDA documentation of patient reported outcomes (PRO) specifies that studies should provide a “test of whether patients understand the items” and establish that “the concepts represented in the PRO instrument’s conceptual framework are confirmed, that the response options and recall period are appropriately comprehended, and that the instrument’s readability is adequate for the intended population.” [4]. The FDA do not specify how their objectives are achieved as different methods may be appropriate in different circumstances, but it is logical that studies evaluating patient understanding of a questionnaire should be carried out after a draft questionnaire has been constructed. That is, there should be two stages in the process of qualitative research: a first stage of domain identification which can be used by the researchers to construct a questionnaire, and a second stage when patients are given a questionnaire to comment on and their comments then can be used to confirm or change the existing wording, including the conceptual framework. Both wording and conceptual framework could differ between patients and researchers.

A 15 item draft questionnaire was constructed on the basis of domains identified in our earlier research [6], reflecting the frequency of problems identified by patients in that earlier study, and with the structure of the questionnaire modelled on the Asthma Bother Profile (ABP) [11]. The ABP is the only asthma scale that was constructed using the two stage process of qualitative research (described above) for mild or moderate people with asthma. The structure of the questionnaire, based on patient preference, is to have key words (e.g., social life, personal life), written in large text, and, for some items examples of what is meant by the key words written afterwards (e.g., visiting friends, walking with friends, talking with friends, going to bars/restaurants and parties). The response options for the Asthma Bother Profile are degrees of bother caused in each of the specified areas of concern. For this scale, the response options were a 7-point scale of how your life was affected with patients asked to attribute whether the aspect of life was attributed to asthma symptoms, asthma medicines except oral steroids, and oral steroids. A global health-related quality of life scale was added at the end of the questionnaire, following the design used by the EQ-5D-5 L where a visual analogue scale (VAS) is added after the initial five questions [12]. Unlike the EQ5D VAS which measures perceived health, we based the 100 point scale on a global quality of life scale (GQoL), which is a 100 point Borg scale of quality of life [13]. Patients were asked to rate their quality of life on average and to separately estimate their quality of life due to asthma symptoms and side effects of medicine.

The aim of this study was to improve this draft questionnaire by presenting it to patients and asking for comments and ways of improving it.

## Methods

### Participants

Patients who were attending a severe asthma clinic in the UK were invited to attend a focus group. Patients were selected so as to recruit both genders, a range of ages and severity assessed by British Thoracic Society (BTS) steps of asthma management (which is defined by treatment) and representative of the population of patients seen in this clinic. There were four focus groups, 16 participants of whom 12 were female, with ages ranging from 24 to 69 years and a mean age of 47 (SD = 13.53). Participants were at BTS steps 3, 4, and 5. More females than males were able to take part in these day-time focus groups.

### Procedure

Patients gave written informed consent to take part in a study on the development of a questionnaire, and completed the questionnaire on arrival. The purpose of the focus group was explained – for patients to help construct a severe asthma questionnaire. Patients were asked specific questions about every element of the questionnaire, including the introductory words, the response scale, and the individual items. For each element the patients were asked to describe what they understood by the meaning of the element and individual words in the element. Patients were encouraged to discuss if and how the element under consideration could be improved, including the balance between different types of item in the questionnaire. The sessions were moderated by one of the authors, observed by another, and changes recommended by patients noted. The recommended changes were then implemented into the next version of the questionnaire which was presented to the subsequent group. All authors contributed to and approved the final version of the questionnaire. The sessions lasted up to 2 h and were audio recorded.

The study was approved by the Plymouth Hospitals NHS Trust, ethical approval number 16/NE/0188, IRAS ID: 207,601.

## Results and discussion

Information gained from patients during the iterative process of questionnaire modification can be divided into four categories.

### Change to the concept

One of the aims of questionnaire development was to provide independent assessments of the impact of asthma symptoms versus the impact of the side effects of asthma medicines on quality of life. Despite iterative changes in wording, we found that although some patients in each focus group could answer the question of relative impact, significant problems arose for some patients and these are noted below.

First, some patients noted the difficulty in identifying asthma medicines from other medicines, and in particular those medicines that were taken to counteract the side effects of asthma medicines. Patients differed as to whether these side effect medicines should be considered asthma medicines or not.

*P5. I have a lot of medication, that counteracts medication that I’m on, but I still consider it my asthma medication, because it’s all for that. So would you include things like that?* 16.20. 20/07/16

*P1. Zopiclone sleeping tablets, because you get, stop sleeping when you’re on 10mg.* 16.25. 20/07/16

*P2. And I know that Zopiclone that I’m now on, does have side effects, and it’s addictive, and I’m now addicted to it.* 19.25. 20/07/16

Second, the issue of deciding between disease versus treatment was perceived as difficult and unfamiliar task.

*P15. It’s actually like making my brain think. Cos I’ve never been asked any of these questions before, in the whole time I’ve been under Consultants and everything, I’ve never been asked any of this stuff.* 14.11. 22/07/16

Third, for patients who had been on long term oral corticosteroids since childhood, many were unaware that this type of medication had side effects.

*P13. It’s very difficult, as you tend to ignore those as life, rather than these are side effects caused by your medication.* 08.05. 21/07/16 PM

*P16. I think what’s interesting here is, I’m not sure what the side effects are.* 25.03. 22/07/16.

Finally, some symptoms may be caused either by the disease or treatment. For example, patients can experience fatigue after an exacerbation requiring admission to hospital (which of particular problem in relation to child care), but it is unclear whether fatigue is a response to the exacerbation, the reduction in oral corticosteroids or simply the stay in hospital.

The conclusion drawn from patient feedback was that it is possible for some patients to make a meaningful assignment of problems to asthma symptoms versus side effects of medicine. However, because in each focus group one or more patients who reported problems in relation to this concept, the conclusion was any assessment of the concept of symptom attribution would not be content valid.

### Changes to recall period

The moderator explained to patients that the aim of the questionnaire was to use it in clinical trials, and that a 2 week recall period is used elsewhere [3]. Patients found a 2 week but not a 4week recall period acceptable.

*Yea, last couple of weeks. Four weeks is really hard. How many people can remember how you were four weeks ago?* 40.50 20/07/17

*P6. I think if you’re going into, if you want detail then two week is about my limit really [laughs].* 09.06. 21/07/16 AM

The use of a 2 week period was supported by a second participant who was critical of other asthma QoL questionnaires used as part of clinical practice that require a 4 week assessment period.

*P4. It’s bad enough when you go to the chest clinic and fill them out isn’t it. Because they say, in the last four weeks how have you been feeling, and I’m thinking, that’s four weeks ago.* 06.30. 20/07/16

However, although patients could report on the last 2 weeks, they felt that this recall period provided a poor description of their experience of asthma.

*P7. It sounds extremely like you are going on textbook asthma, but asthma is never textbook.* 31.17 21/07/17 AM

Patients would like to be able to express how their asthma affected them over a longer time period.

*P7. The thing is, if you want a genuine direct answer, and you want the best results possible, you probably would have to break it down into months.* 07.28. 21/07/16 AM.

*P3. Just do 3 sections and say, what’s it been like in the last 12 months, and then particularly the last 6 months, and then particularly in the last month.* 40.00. 20/07/16

Patients indicated that their symptoms and quality of life varied with the season.

*P13. I know exactly when I will be ill, I know that I really struggle with the mould season in the autumn, and that in the spring, between February and May is my good time of year.* 11.05. 21/07/16 PM

*P7. Think seasons’ll be good because people are going to be affected, as you say depending on, what their aller [sic], you know the allergic asthmatics. So if you’re allergic to the funguses in the autumn, you might be worse in the autumn, you’ve got the spring flowers, you’ve got the summer grass.* 06.40. 21/07/16 AM

Patients also indicated they would like to assess their quality of life on average.

*P3. You have a chance at remembering how you felt on average, because you can have bad days and you can have good days.* 41.00. 20/07/16

In order to satisfy the competing demands of a clinical trial and the patients’ desire to provide a more holistic description of their asthma, one solution explored was to ask patients to rate all items on a 2 week period but also, on a separate page, to assess global health-related quality of life during the four seasons of the year. The additional questions about the four seasons (i.e., four global questions, one for each season) was found acceptable by patients in the focus groups. After the focus groups were completed, an international group of clinicians advised that seasons do not apply in some countries. In order to satisfy patient preference without referring to seasons, four global season questions were replaced by one global question to assess quality of life during the last 2 weeks and two global questions to assess quality of life during the best and worst months of the year. This solution was found acceptable by a small group of patients in clinic.

### Changes to the response scale

The initial response scale for the 15 items consisted of a 7-point scale of how a patient’s life was affected, with patients asked to write a number in a box to show whether the aspect of life was attributed to asthma symptoms, asthma medicines except oral steroids, and oral steroids.

As reported above, patients found it difficult to attribute cause to symptoms, and several felt that tick boxes were preferable to writing numbers. Because of patient feedback, subsequent versions of the response scale were simplified to a tick box scale.

The meaning of the words used in the response scale was discussed. Patients interpreted the word ‘restricted’ – the word used in the original format of the scale – in a sense equivalent to ‘I am unable to’ rather than in terms of a limitation that varied in degree, and patients suggested the term ‘difficult’ instead.

*P7. difficult I would find better because restricted, you know, means there’s a limit, you know, difficult could be to any degree.* 43.20. 21/07/16 AM

Following discussion, the final questionnaire has a response scale with a 7 point difficulty scale: very, very difficult (worst possible); very difficult; difficult; moderately difficult; slightly difficult; very slightly difficult (just noticeable); no problem, with quantifiers taken from earlier research on the use of quantifiers in category rating scales [13]. This final version was confirmed separately with patients in clinic.

### Changes to the wording, structure and content of the items

Although the term exacerbation was not used in the questionnaire, when the moderator suggested that sometimes health professionals used words that patients did not like, patients gave the following response.

*P4. And stop calling it, ex…*

*P1. Oh exacerbation (Mispronounced).*

*P4. Nobody calls it that! We all call it asthma attack.* 57.30. 20/07/16

Patients suggested several changes to the items and wording of the questionnaire and this was done iteratively. In an early version of the questionnaire there was a question about family life. Patients were asked to rate the difficulty of their asthma and its treatment associated with “My family life. For example: child care, family responsibilities”. Discussion indicated that there were two very different aspects of family life: that of the patient and that of the patient’s family. The impact of family is much pronounced in severe asthma because of the frequency of exacerbations and hospitalisation.

*P6. Yea it should be recognised that peoples’ families are taking on a burden.* 42.53. 21/07/16 AM

As a result, the final version of the questionnaire has two items relating to family life (see Table 1).

**Table 1:** Table showing the principal item changes between the first and final version of the questionnaire

Initial questionnaire item(s) presented to focus groups	Final questionnaire item(s) after iterative changes
3. I am restricted in my family life. For example: child care, family responsibilities.	6. My family life – how it affects me. For example: caring for children, family responsibilities. 7. My family life – how it affects others. For example: others taking time off work, problems with childcare, family members becoming upset.
10. I worry about my asthma and treatment in the future. For example, asthma getting worse, long term side effects of medicines.	11. Worry that asthma may get worse. For example, medicines no longer help, more frequent attacks. 12. Long term side effects of medicines. For example, cataracts, diabetes, bone fracture.
12. My sleep is disturbed. For example, difficulty going to sleep, being woken very easily, waking often at night.	14. Problems at night. For example, difficulty going to sleep, being woken very easily, waking often at night.
13. I dislike the way I look. For example, I don’t like my weight, my skin bruises easily. 15. I get embarrassed. For example, I don’t like using my medicines in public, I don’t like having asthma symptoms in public.	15. The way I look. For example, my weight, my skin bruises easily, using medicines in public, other people judging me.

In the draft questionnaire, there was one item concerning worries about medicines. Discussion with patients showed that there were two distinct concerns: that asthma medicines would become less effective over time, and that the medication would produce side effects.

*P2. Omeprazole, if you’re on it long term, can be quite damaging.* 19.00. 20/07.17

*P1. Steroids used to improve my life, I thought they were wonderful because I was able to do things, and now I am at the stage where I am taking a lot of steroids every day and I can’t. I struggle with the stairs, I struggle to have a shower, you know it’s, it’s crap.* 24.11. 20/07/17

*P1. I used to think of the benefits, now I think of the side effects because I’m reaping them.* 14.40. 20/07/17

Patients therefore felt that the item on medication should be split in to two to reflect two very different concerns about medication (see Table 1).

*P6. Should there be somewhere about the more longer term problems, like the cataracts and the diabetes, so that you’ve got a record all these. 53.41. 21/07/16 AM*

Although patients felt that some items should be split, they felt that two items could be combined, namely the items on weight and on embarrassment, as having two items provided excessive emphasis on this aspect of quality of life deficit.

*P9. When you out “getting anxious” umm, it kinda combines with “the way I look” also. What I’ve just said you know, people pre-guessing exactly what is wrong with you, because you’re not in a wheelchair.* 52.11. 21/07/16 AM

Another patient reflected that they felt self-conscious as a result of having asthma, which stemmed from the public’s lack of understanding of the disease.

*P8. There’s a stigma about asthma isn’t there.* 52.35. 21/07/16 AM

In addition to changes to the structure of the questionnaire, patients also made changes to wording within items. Patients pointed out that a question about sleep did not ask about periods during the night when the patient was unable to sleep.

*P16. What about night times?* 28.20. 22/07/16

*P16. Well sometimes, I have er, so I don’t disturb the wife, I go downstairs, you know I use my pumps and all that, but I go downstairs, so I don’t wake her up. You know, I do get umm tight chest night times, and I’m woken up.* 28.32 22/07/16

*P16. If I’m tired, you know, and I wanna go to sleep, and you can’t go to sleep, and sometimes it does get to you, you know. Well upset I suppose.* 29.16 22/07/16.

*P14. Mines worse if I’ve got a chest infection as well, and then I’ve got my nebuliser right beside my bed.* 29.32 22/07/16.

The word ‘night time’ was therefore used with sleep as an example of what happens at night.

Other changes involved amplification. For example, in the original scale there was an item ‘I get tired’. The final version is “Getting tired. For example, feeling tired for no reason, waking in the morning feeling tired.”

Patients were asked to compare the final version of the SAQ with other questionnaires completed in clinic. Responses were positive, none negative.

*P14. The one in the clinic feels like, it’s very kind of like medical, and have you done any exercise? Have you lost sleep? Umm, it’s kind of, they are just trying to establish how bad your asthma is at that point before you go and see the Consultant. Whereas these ones feel more, how your quality of life in general is, which I’ve never been asked about. Which is something which really frustrates me and this is the first time I’ve been asked about it.*

14:47. 22/07/16

## Conclusions

This study demonstrates that more than one type of qualitative research is need to achieve content validity. An initial questionnaire was written on the basis of interviews with 23 patients [6]. That initial questionnaire was written by a team that included a psychologist who had constructed ten published scales of which four relate to respiratory medicine, a physician who had constructed a questionnaire for COPD patients, and a respiratory specialist leading a severe asthma clinic. Despite the expertise of this group, feedback from patients who evaluated that questionnaire created a final version that was not only radically different from the initial, but also differed in concept.

Patients who are prescribed OCS have poorer health-related quality of life [14], and greater health resource utilisation possibly attributable to side effects of OCS [15]. Interest in assessing the side effects of OCS is motivated by the development of new biologic agents that can control inflammation in asthma and other inflammatory diseases and therefore reduced the burden of OCS and its side effects. Feedback from patients showed that whereas some could identify whether a symptom was caused by OCS or asthma symptoms, there were four problems that could make the attribution of cause difficult for patients. These problems are compounded by that fact that exacerbations lead to increased OCS exposure, but exacerbations are defined by worse asthma symptoms. That patients have difficulty in attributing cause to disease versus side effects of OCS has been reported in other disease areas [16], and attribution of cause in other contexts shows that people often make incorrect attributions [17]. Because of the number of patients having problems with the attribution of cause (at least one in each focus group), the original intention to provide separate measures of asthma symptoms and medicine side effects was abandoned.

Although the initial qualitative interviews led to a 15 item questionnaire, the outcome of the second phase of qualitative research was to produce a 16 item questionnaire. Patients changed the structure and balance of the items. Two of the original items were split into two creating four out of the original two items, but two of the original items were combined. Patients made other changes to content of the items, and they also made changes to the response scale. Although the unsuitability of the term ‘exacerbation’ has been noted before [18], we found problems with additional words where patient and health professional interpretations were slightly different.

The SAQ is designed for clinical trial use in asthma where an assessment period of 2 weeks is appropriate for underlying biological changes. A 2 week assessment period is applied in a commonly used asthma specific questionnaire [3]. Severe asthma is characterised by frequent exacerbations, and patients were clear that any 2 week period of assessment does not capture the full impact over the course of a year. The FDA documentation states that the recall period should be appropriate for “The population, disease state, or application of the instrument” [4]. The problem we faced was that a 2 week recall period is appropriate for the intended application of a clinical trial, but not for the patients’ own perceptions of the disease state. We therefore adopted a compromise where 16 items and one global scale refer to a 2 week period, but patients are also asked to rate their global quality of life in their worst and best months. However, it should be noted that a deficit measured in any 2 week period therefore cannot be generalised over a year. The problem of generalisation is important for health resource allocation. The EQ-5D-5 L [12] assesses health impact during the day of assessment. A very large sample would be needed to randomise out the variation of asthma that occurs in severe asthma over a period of a year. In addition, the impact of asthma can vary by seasons. Thus, point measurement of a variable condition has the potential for error.

The recall period of 2 weeks is appropriate for a clinical trial, but patients also have a narrative they want to tell the clinician. Time estimation is influenced by interest and enjoyment [19, 20]. The global questions at the end of the questionnaire contribute to a more positive experience of questionnaire completion which, on the basis of reports from patients in this study, is often lacking in other questionnaires. The SAQ takes between three and 6 min to complete and produces two scores: an aggregation of the 16 domain relevant items to produce an SAQ score for the last 2 weeks, and a SAQ-global score produced from the single global estimate of quality of life over the last 2 weeks. The worst and best month scores can be used for clinical purposes but the research use (for example, in detecting response shift) has not been determined. The questionnaire can be downloaded from www.saq.org.uk.

**Fig. 1:**
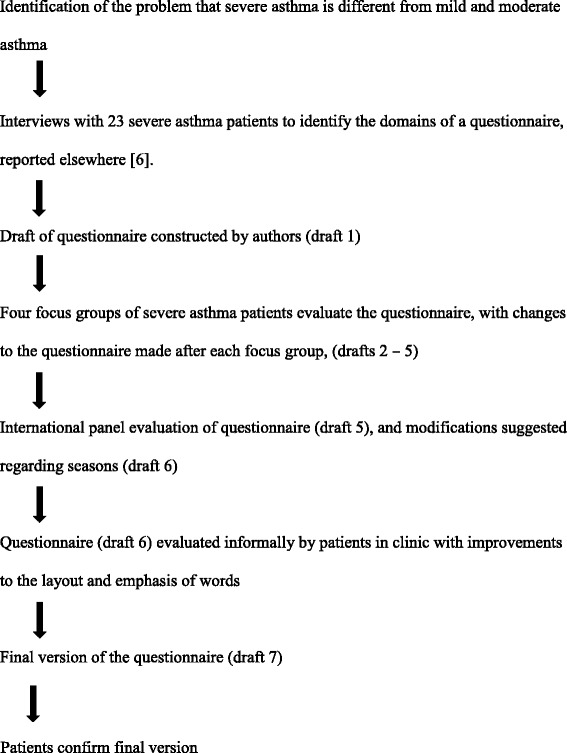
The process of questionnaire development of the SAQ

Edwards et al. [1] list problems that can reduce the content validity of a scale. Qualitative research can help avoid those problems. A diagrammatic summary of the steps taken in content validation is shown in Fig. 1. In our earlier study we showed that existing asthma specific scales failed to include items relevant to the burden of treatment. In the study reported here, patients commented on a draft questionnaire and these comments then led to alterations to the concept; that some items should be split in two whereas others should be combined, that changes should be made to the response options and time periods and changes to wordings of individual items. Finally, this study has shown the considerable benefits of treating patients as partners in the process of questionnaire. A considerable body of research shows how the language used by health professionals differs from patients [18]. Engaging patients as partners can produce, what patients perceive as an improved questionnaire, compared to treating patients only as sources of information.
